# Impact of Outdoor Play Structures on Moderate to Vigorous Physical Activity in Children during Recess: A Comparative Study

**DOI:** 10.3390/children11070828

**Published:** 2024-07-07

**Authors:** Maria Fernanda Fuentes Diaz, Martin Sénéchal, Danielle R. Bouchard

**Affiliations:** 1 Faculty of Kinesiology, University of New Brunswick, 90 Mackay Drive, Fredericton, NB E3B 5A3, Canada; ma.fernandadiaz@unb.ca (M.F.F.D.); martin.senechal@unb.ca (M.S.); 2 Cardiometabolic Exercise and Lifestyle Laboratory, 90 Mackay Drive, Fredericton, NB E3B 5A3, Canada

**Keywords:** MVPA, school break time, heart rate, outdoor activities

## Abstract

Background/Objectives: It is believed that outdoor play structures lead to more physical activity for kids during school recess. However, the intensity of this activity remains unknown. This study explored whether access to outdoor play structures during recess interferes with children’s physical activity levels. Methods: Forty-one children (8–10 years old) accessed play structures during the afternoon recess but not in the morning for one entire week. To control for temperature differences, the same number of participants from another school who did not access playground structures were invited to participate. Moderate to Vigorous Physical Activity (MVPA) was determined using heart rate reserve. Heart rate was recorded using the Fitbit Inspire 2 (San Francisco, CA, USA) for at least three full school days. Wilcoxon signed-rank and Mann–Whitney U tests analyzed within- and between-group differences. Results: The findings show no difference in MVPA when accessing or not accessing outdoor play structures, both within groups [(n = 37) median (25th–75th) 16 min (7–30) vs. 14 min (5–22)] and between groups [(n = 22) 16 min (7–26)]. The weekly MVPA for all participants (n = 59) [172 min (117–282)] was the strongest variable associated with MVPA during recess [t(df) = 5.40 (38), 95% CI 0.04–0.09, *p* < 0.001]. Conclusion: accessibility to outdoor play structures does not increase MVPA during recess in children aged 8 to 10. Therefore, schools may need various options for children to play during recess, allowing them to accumulate MVPA.

## 1. Introduction

For optimal health benefits, children aged 5 to 11 should accumulate at least 60 min of moderate to vigorous physical activity (MVPA) per day [1]. However, only about one-third of children meet the physical activity recommendations globally [2]. The main factors affecting physical activity levels among children are age and sex [3], but temperature and precipitation also influence the level of activity performed outdoors [4,5].

Schools are uniquely positioned to positively influence physical activity levels, as they reach most children and youth, regardless of gender, race, ethnicity, or family circumstances [6]. Additionally, time spent at school, specifically during recess, provides children with the opportunity and space for physical, social, and emotional development [7,8]. 

Physical activity levels during recess are influenced by factors such as equipment availability, facilities, space, policies at school, and support [9]. During recess, children can typically engage in free play or use play structures. Free play is a critical element of a healthy lifestyle for children [10], and it is usually performed outdoors, where children exert energy in a freely chosen, fun, and unstructured manner [11]. On the other hand, play structures are built and designed to offer opportunities for children to have predetermined sequences of play [12,13]. Although attractive, play structures are costly, and for many, no budget is planned for these when a school is built [14]. Moreover, play structures increase the risk of injuries and offer limited activities [15,16]. 

Some evidence points out that the absence of outdoor play structures does not necessarily negatively impact children’s physical activity levels, since free outdoor play still constitutes a solid opportunity to be active [10,11]. Although no study has rigorously compared access vs. no access to play structures, some evidence suggests there is no advantage of play structures. For instance, Wood et al. tested the differences between playgrounds and open spaces with 25 children aged 8–9 and reported an advantage of free play in open space [17]. This study reported that 61.6% of the variance in MVPA was due to the playing environment. Participants engaged in 40% more MVPA when playing on the field compared to the playground. However, one of the main limitations was that they did not describe if outdoor play structures were present in the playground; in addition, they used accelerometers to measure physical activity only during the final two days of each week, which might not be fully representative of overall weekly activity, and the novelty of having the accelerometer attached to the participant’s hip could have introduced some bias toward normal children’s behavior [17]. 

In the present study, outdoor play structures were part of the comparison, and watches were used to record heart rate and measure physical activity intensity. Watches were selected so children felt more comfortable wearing them without introducing bias, favoring adherence to their use [18]. Data collection lasted seven days, including five school days, to represent a child’s whole week accurately. These specifications address the limitations of the previous study and will help add new findings to the current body of evidence. Moreover, findings from this study could inform school policies or school-based interventions aimed at promoting physical activity among children.

In this pilot study, we aimed to assess whether access to outdoor play structures during recess interferes with children’s MVPAs and to test whether potential factors influence the level of physical activity in children during recess at school. The secondary outcomes were minutes of MVPA during other times of the week and variables associated with MVPA during school recess. We hypothesized that the number of minutes of MVPA among children aged 8–10 would not be different whether they accessed or did not access outdoor play structures during recess time.

## 2. Materials and Methods

### 2.1. Setting and Participants

This project was approved by the Research Ethics Board of the University of New Brunswick and is on file as REB 2022-104. Approval was obtained from the Department of Education and school districts. Principals of public schools with and without play structures were contacted, and those who expressed interest were formally invited to participate. Then, the principal of each school informed parents and staff of the study. After this announcement, parents received an envelope containing all the information, forms, and questionnaires over the following week. To be in the study, the consent and assent forms had to be returned to the teacher and researcher. The inclusion criteria were children aged 8 to 10 years who provided written assent and also written consent from their parents or guardians. Parents or guardians witnessed their children’s written informed assent. Exclusion criteria included children whose parents reported them being under heart, asthma, or blood pressure medication. 

Two public schools participated in the study. Participants from the experimental school (ES) had no access to play structures during the morning recess but access to them during the afternoon recess. Since it has been reported in the literature that temperature might affect physical activity when performed outdoors, another school was recruited to allow for data collection at the same time of the day. Participants from the control school (CS) had no access to play structures during any recess times. Since the duration of recess was different in the morning and afternoon, only the first 20 min of each recess time were analyzed.

Based on available resources and to collect data during the same period at both schools, 82 children from the two schools were invited to wear a Fitbit (Inspire 2, San Francisco, CA, USA) for seven consecutive days. Researchers charged and set up Fitbits a day before they were distributed to principals. Researchers explained to principals and teachers how to distribute and wear the Fitbits. Then, the principals and teachers distributed the Fitbits to the children who had signed and returned the consent and assent forms. Teachers instructed children to wear Fitbits all the time for a week, and a letter was sent to parents explaining that their children were participating in the study and some recommendations on how to wear the Fitbit. After seven days, teachers and principals collected the Fitbits back and returned them to the research staff.

Before the observation week, parents were asked to answer a questionnaire with their children’s information, as follows: age, sex, grade, and if the child was registered in organized sports during the academic year, as well as their own sex, age, education level, income, and employment. Parents also reported their physical activity via the Godin–Shepard Leisure-Time Physical Activity Questionnaire [19] and weekly resistance training frequency. Those who reported a leisure score index ≥ 24 were classified as active, while those who reported <24 were classified as insufficiently active [19].

### 2.2. Moderate to Vigorous Physical Activity

Children from both schools wore the Fitbit on their nondominant wrist 24 h a day during the same seven days (Friday to Thursday). Time spent at moderate-to-vigorous intensity was measured via heart rate using a wrist-worn Fitbit (Inspire 2, San Francisco, CA, USA). Fitbit is one of the most popular commercial wearable activity trackers that allows for recording heart rate using a noninvasive photoplethysmography technique [20,21]. In pediatric populations, Fitbit has shown to record accurate heart rates compared to electrocardiography monitoring for children at rest and performing light activities (r = 0.99; average bias of −0.05 bpm, 95% CI: 2.454–2.43 bpm) [22], as well as heart rate chest straps, with an absolute percent difference of 6.9% (r = 0.84) [23].

Moderate to vigorous physical activity was defined as an activity performed at a minimum of 50% of the heart rate reserve [24]. The following formula was used to estimate the minimum heart rate to be considered as moderate intensity [25]:

(200 − resting heart rate)*0.5 + resting heart rate

Heart rate reserve was chosen because it effectively estimates heart rate thresholds appropriate for promoting health and fitness in school-age children, as it accounts for individual resting values [26]. The maximum heart rate was estimated to be 200 for all participants [27]. Resting heart rate was determined as the lowest record of each day during the sleeping time (10:00 p.m. to 7:00 a.m.). When sleeping time was unavailable, resting heart rate was determined by the lowest record of the day during waking time. Fitbit Intraday Heart Rate Service and a Python were used to extract data (Appendix A. Code for data extraction). Only children who provided complete data during school time for at least three days were included in the analysis [28,29]. Missing information was replaced with the average of the recorded valid days. Data were analyzed using the following three steps: First, the average heart rate for each minute recorded and wearing time during the school day was estimated. Then, heart rate records above 50% of the heart rate reserve were converted to minutes of MVPA per day. Records were taken during the daytime (7 a.m. to 10 p.m.), school time, and morning and afternoon recess times following the school schedule provided by principals. Finally, the results from each day were summed to estimate the total weekly minutes of MVPA for each child during the following times: (1) week—daytimes of seven consecutive days of data collection; (2) weekdays—daytimes from Monday to Friday; (3) weekend—daytimes of Saturday and Sunday; and (4) school times—based on the weekly schedule provided by principals.

### 2.3. Weather Conditions

The following information was collected from Environment Canada’s weather website (www.weather.gc.ca/canada_e.html, accessed on 6 July 2023) to understand the context in which data were collected: temperature (T°), precipitation (mm), and relative humidity (%). The mean temperature, precipitation, and relative humidity were recorded during the same seven consecutive days when children wore Fitbits. The weekly average of each weather condition was calculated.

### 2.4. Dimension of Play Space

The dimensions of each school’s playground area were measured on-site using a measuring wheel (m). Then, the available play space per child on the playground (m^2^) was obtained by dividing the available playground area for each school by the number of children at the school, as reported by school principals.

### 2.5. Data Analysis

Three analyses were performed: (1) within groups—ES (n = 37), nonaccess vs. access, different time of the day; (2) between groups—ES (n = 37) vs. CS (n = 22), same time of the day to control for temperature differences; and (3) between groups—ES, access (n = 22) vs. CS, nonaccess (n = 22), matched by the children’s age and sex. The significance level was set at 0.05, and all analyses were performed using SPSS version 28 (IBM SPSS, Chicago, IL, USA).

The demographic characteristics of the participants and the weather are reported as the median and interquartile ranges or frequencies and percentages. This approach was selected given the small sample size. A Kolmogorov–Smirnov test determined that the primary outcome residuals were not normally distributed. A Wilcoxon signed-rank test was computed for the within-group analysis to compare time spent engaging moderate-to-vigorous intensity when accessing outdoor play structures (n = 37); a Mann–Whitney U test was used to analyze the difference between groups (n = 59). A quantile regression analysis of factors potentially associated with time spent engaging in moderate-to-vigorous intensity among children during recess was performed for between-group analysis, adjusting for children’s age, sex, weekly MVPA level (excluding recess time), and parents’ physical activity levels.

## 3. Results

A total of 41 children and their parents from each school were recruited, but only n = 37 from the ES and n = 22 from the CS had valid data related to the primary outcome. The average temperatures during the five school days when data were collected were 11.5 ± 4.3 °C and 14.8 ± 2.7 °C during the morning and afternoon recess times, respectively. The average precipitation was 7.9 ± 10.4 mm, but it occurred after school hours. There was no precipitation during recess; thus, the primary outcome was not affected. Finally, the average relative humidity was 79.8 ± 1.7%.

On average, children from the ES had 15 m^2^ and 13 m^2^ of spaces to play in during the morning and afternoon recesses, respectively. The spaces consisted of both vertical features (i.e., manufactured features) and horizontal features (i.e., markings and surfaces designed for activity). In contrast, children from the CS had 25 m^2^ of space to play in during recess. They had access to an open space for the entire duration of the recess, which consisted of grassy areas surrounded by trees.

Table 1 shows the demographic characteristics of the children with valid heart rate monitoring data and their parents. The children’s median age was eight years old, 53% of the sample was female, and 62% participated in sports during the academic year. The reporting parents were mainly women (70%). Parents of the children from the CS were older than those from the ES, but no significant differences existed in their incomes, education levels, or employment situations.

**Figure 1:**
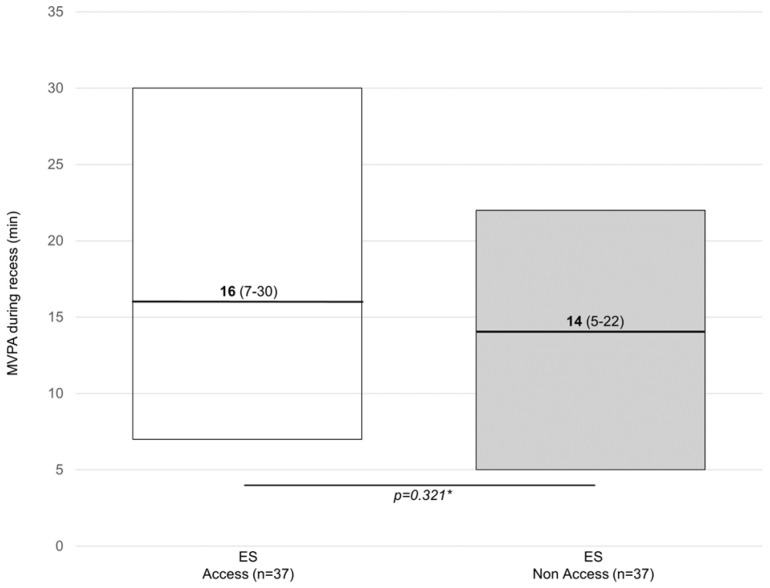
Weekly minutes of MVPA during recess among children from the ES. Data are presented as the median (25–75 IQR). ES = experimental school; CS: control school. * Wilcoxon signed-rank test.

**Figure 2:**
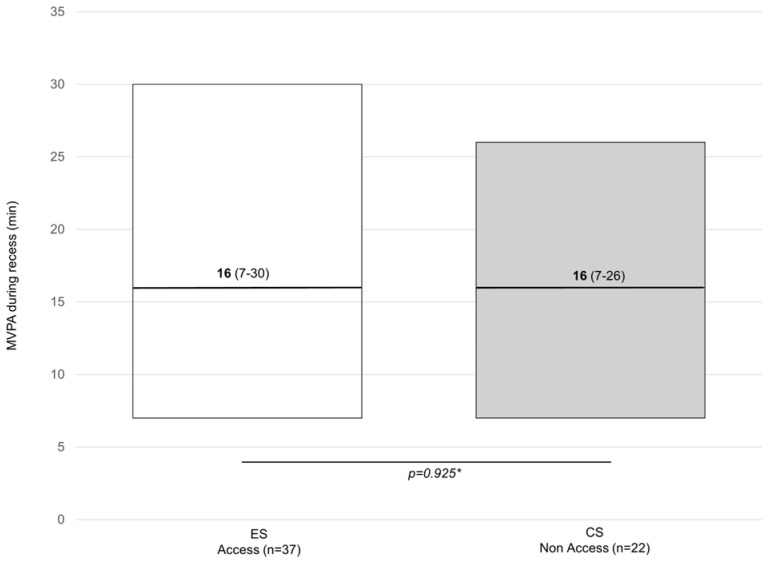
Weekly minutes of MVPA during recess among children from the ES vs. CS. Data are presented as the median (25–75 IQR). ES = experimental school; CS: control school. * Mann–Whitney U test.

**Figure 3:**
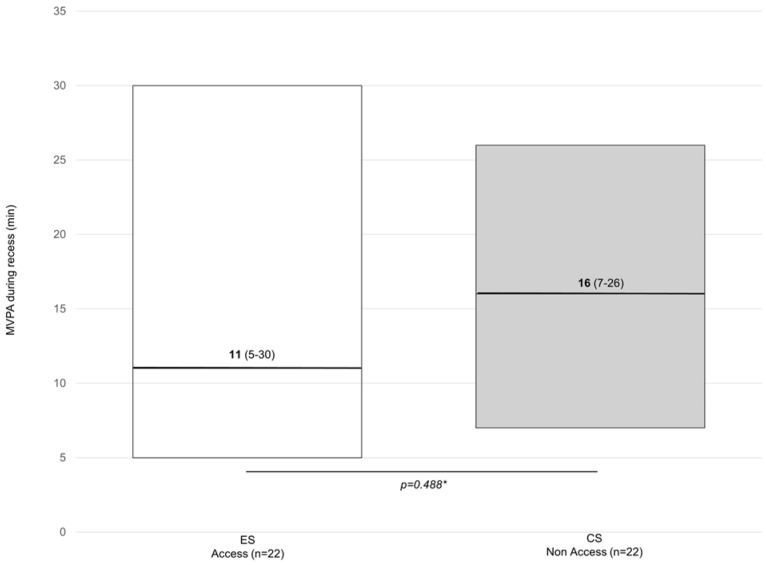
Weekly minutes of MVPA during recess among children from the ES vs. CS, with samples matched by sex and age. Data are presented as the median (25–75 IQR). ES = experimental school; CS: control school. * Mann–Whitney U test.

Figure 1, Figure 2 and Figure 3 show the weekly minutes of MVPA during recess. Figure 1 shows the results from the within-group analysis using data collected at two different times of the day (i.e., morning and afternoon). Figure 2 shows the results from the between-group comparisons controlling for temperature (same time of the day). Figure 3 shows data from matched samples by sex and age. Out of 100 min analyzed over the school week (five days for 20 min each recess), no difference in MVPA was observed when accessing or not accessing the outdoor play structures within groups (n = 37) [16 min (7–30) vs. 14 min (5–22)] or between groups (n = 22) [16 min (7–26)].

Table 2 displays the minutes spent at moderate-to-vigorous intensity during different weekdays and recess times. No significant difference was observed between the groups. For example, children from the ES (having access to outdoor play structures) spent 173 (104–266) minutes at moderate-to-vigorous intensity vs. 171 (130–298) minutes for children from the CS. Most children (81%) in the sample did not meet the weekly physical activity guidelines of 60 min at moderate-to-vigorous intensity per day.

The quantile regression (n = 59) revealed that access to play structures does not predict the median number of minutes spent at MVPA during recess, nor does children’s age, sex, or parent’s physical activity level (see Table 3). However, total weekly minutes (without the recess MVPA) of physical activity was strongly associated with the level of activity during recess time [t(df) = 5.40 (38), 95% CI 0.04–0.09, *p* < 0.001]. 

## 4. Discussion

This pilot study aimed to assess whether access to outdoor play structures during recess impacts children’s levels of physical activity. Secondary outcomes aimed to examine the number of minutes that children spent at MVPA at other times during the week, as well as the potential factors that influence children’s MVPA. The results of this study suggest that play structures do not impact children’s physical activity levels, as there was no difference in MVPA when accessing or not accessing outdoor play structures, nor when controlling for temperature, sex, or age differences.

Inverse to what was observed in the current study, a previous study reported 40% more time at moderate-to-vigorous intensity when children (age 8.6 ± 0.3) were involved in free play compared to a playground [17]. However, what they call a “playground” consisted of concrete areas surrounded by school buildings, whereas trees and bushes surrounded the free play area [17]. This speaks to how comparing studies is complex as fields and structures vary from one school to another and likely even more among countries. Nonetheless, another study conducted by Berg showed that children tend to be more active if the area includes grass [30], which was not the case in the schools included in this study. Finally, another study also found that physical playground features were not associated with physical activity at any intensity when evaluating 128 children aged 9–10 years old from eight schools [31]. 

The existing literature in this area may help to explain our findings. Perhaps children in our study did not achieve a more moderate to vigorous activity when accessing the outdoor play structures because the available space was similar. One study reported that children are more active in spacious environments independently of structures [32], and another suggested that children can be active in a poorly resourced environment, as they can engage in locomotive activities associated with moderate to vigorous intensity [33]. These findings suggest that the presence of play structures alone might not determine the intensity of physical activity among children during recess. 

Given the main results of this study, it is worth asking why one would invest in outdoor play structures in schools. Play structures are unnecessary if the goal is to increase MVPA. However, a more robust study design and larger sample size are needed to confirm these observations. Not having access to playground structures during school recess may even have advantages. For example, falls from playground equipment are the most prominent single hazard pattern associated with playground use [16], with an annual average of 5222 hospital stays in the US [34] and related healthcare costs estimated to be CAD 106 million in Canada [35]. Also, most school staff perceived a lack of staff resources to supervise children using the playground structures [36]. Another argument for prioritizing recess settings without playground structures is that the cost is usually not publicly funded [15]. On the other hand, outdoor play structures may offer other perceived benefits unrelated to physical activity level. For example, school staff has reported that play structures have extrinsic values of peer relationships and social development for children [36]. Another study conducted with 9–12-year-old children described that playgrounds support children’s autonomy, competence, and relatedness which might not be observed in a free play setting [37].

Children in the current study were relatively inactive, with only 18.5% meeting the weekly MVPA recommendation. It is difficult to observe a difference between the two settings if neither of the settings increases the time spent at moderate-to-vigorous intensity during recess. Participants exhibited a moderate to vigorous intensity during approximately 17% and 18% of recess time whether having access or not to play structures, which is below the percentage reported by Wood et al. [17]. However, it is possible that the low physical activity levels observed in our study sample are a COVID-19 effect that could be attributed to behavioral patterns adopted during the pandemic that persist even without pandemic restrictions. For example, Burkart et al. reported that, from 2018 to 2019, children decreased their MVPA by 8 min, but for the next year, they had a decrease of 16 min [38]. In addition to these findings, Yelizarova et al. reported that, in 2020, 47.0% of boys and 33.4% of girls of school age reached the recommended MVPA compared with 35.3% and 17.9%, respectively, in 2021 [39]. 

Despite a low proportion of the sample meeting the physical activity guidelines, our results suggest that children’s activity levels during recess are associated with overall physical activity. This suggests that every opportunity during the day is important to contribute to children’s overall movement. 

This study and the literature on the subject raise the question of the purpose of recess. According to Ramstetter et al., recess is a crucial time that children can rely on to freely discover, undertake play challenges, explore their senses, and make independent play decisions away from the confines of classroom walls, restrictive rules, routines, and regulations [8]. This suggests that recess should be a time for noncurricular activities. However, other authors, such as Burris and Burris, suggest that recess is an excellent opportunity to contribute to children’s overall movement [7]. Based on our findings, this contribution is relatively small. Given the association between total and recess-based moderate to vigorous activity, it is crucial to promote strategies to increase physical activity during recess, such as markings, zoned playgrounds, the addition of loose equipment, planned activities, staff involvement, and incorporation of grassed areas and green spaces [40,41].

Additional strategies should also aim to increase physical literacy, as evidence showed that children who met the Canadian physical activity guideline of 60 min of daily MVPA displayed higher physical competence, motivation, and confidence in physical literacy domain scores [42]. It is possible that children would spend more time at moderate-to-vigorous intensity during recess when accessing play structures if they were more physically literate. Another strategy is to increase outdoor time among children, as each additional hour per day spent outdoors has been associated with an extra seven minutes of MVPA [43].

This pilot study shows that children who accessed play structures during recess did not have a higher level of moderate to vigorous physical activity than those who only had access to an open space to play. In the context of schools in which these play structures are not always available (mainly because they are not included in the budget when a school is built or renovated), it is important to highlight the importance of free play as an adequate alternative to promote physical activity among children during school recess. In addition, it is essential to encourage physical literacy in all school-aged children, as it could help kids take advantage of all resources available. Moreover, it will help them participate in more complex activities as they age. Parents and school staff should reflect on their beliefs about outdoor play structures at school, as those might influence their usage and promotion in the school context. 

One of the strengths of this study is the use of heart rate reserve to estimate the intensity of physical activity. Another strength is the three different analyses performed within and between groups, naturally adjusting for differences in the setting and temperature. However, we also acknowledge several limitations, starting with the small sample size that prevents the generalization of the results to larger populations. Second, data were collected for only two 20 min recess times during a week in the fall, which might not fully represent all-year activity. Third, it is possible that even if children had access to play structures, they might not have used them. Future studies could address this limitation by conducting observational studies in addition to an objective measure of physical activity. Observational approaches could also provide a more precise description of the play structures’ characteristics and how children use them, given the high variability of play structures and space dimensions from school to school. Additionally, future studies should include a more detailed description of the proportion of schoolyard space dedicated to play structures vs. open space. Finally, the current study focused only on MVPA. Still, different intensities and types of activities would be worth exploring, as play structures could offer other benefits that fall outside of the purpose of this study.

In conclusion, findings from this study corroborate the initial hypothesis that access to outdoor play structures does not increase MVPA during school recess for children aged 8 to 10. Our findings question the need for outdoor play structures to increase MVPA during school recess. Strategies should include various options that allow children to play freely and still accumulate MVPA. Further research is needed to test strategies to increase MVPA during school recess.

## Figures and Tables

**Table 1:** Demographic characteristics of the participants.

	ES (n = 37) Nonaccess: Morning Access: Afternoon	CS (n = 22) Nonaccess: Both Times
Children		
Age (years)	8 (8–9)	8 (8–9)
Sex (male)	17 (46)	11 (50)
Registered sports (yes)	24 (65)	13 (59)
Parents		
Age (years)	40 (38–44)	37 (33–39)
Sex (male)	13 (31)	5 (23)
Total household income (CAD ≥80,000)	20 (54)	14 (63)
Marital status (married)	31 (86)	22 (100)
Education (college or above)	35 (94)	14 (63)
Employment (full-time)	32 (62)	17 (77)
Leisure time score (≥24: active) *	35 (15–48)	37 (23–56)
Resistant training (times per week)	0 (0–2)	2 (0–4)

Data are reported as the median (25–75 IQR) or N (%). * Only 31 and 13 parents from the experimental school and the control school, respectively. ES = experimental school; CS: control school.

**Table 2:** Minutes of MVPA.

	ES (n = 37)	CS (n = 22)
Minutes of MVPA		
Week (7 consecutive days)	173 (104–266)	171 (130–298)
Weekdays (Monday–Friday)	120 (89–225)	125 (100–247)
Weekend (Saturday–Sunday)	17 (7–46)	31 (21–53)
School time (5 school days)	80 (50–132)	81 (46–148)
Meeting aerobic guidelines (60 min/day)	7 (19)	4 (18)

Data are reported as the median (25–75 IQR) or N (%). ES = experimental school; CS = control school.

**Table 3:** Quantile regression of MVPA during recess (n = 59).

	Coefficient	95% CI	*p*-Value	Model Statistics
Children *	20.92	−37.45–79.31	0.47	Pseudo R square = 0.18; MAE = 8.64
Access to play structures (Yes)	−1.79	−11.06–7.47	0.69	
Age (years)	−1.95	−9.04–5.15	0.58	
Sex (male)	7.96	−0.71–16.63	0.07	
Weekly MVPA without recess (minutes)	0.06	0.04–0.09	<0.001	
Parent’s physical activity (score)	−0.01	−0.21–0.18	0.85	

Data are reported as the median (25–75 IQR) or N (%). * Only 31 and 13 parents from the experimental and control schools, respectively.

## Data Availability

All relevant data are within the manuscript.

## Appendix A

Python Code for Data Extraction

Participant_Code = ‘000_00’

Group = x

import pandas as pd

import numpy as np

import os

import tkinter as tk

from tkinter import filedialog

root = tk.Tk()

root.withdraw()

Path = filedialog.askdirectory(title = “Select a directory”)

os.chdir(Path)

allFilesArray = os.listdir(Path)

#--------------------------------------------------

for file in allFilesArray:

 os.chdir(Path)

 File = file

 Data_Frame = pd.read_excel(File)

 #Create the last row, you must comment on the following fragment if you want to see the graphs

 lastIndex = Data_Frame.index[−1] + 1

 time = Data_Frame.at[Data_Frame.index[−1],‘Time’]

 year = time.year

 month = time.month

 day = time.day

 newDate = pd.Timestamp(year,month,day,22)

 Data_Frame.loc[lastIndex+1] = [newDate,np.NaN]

 #----------------------------------------------

 Date_Time = Data_Frame[‘Time’].astype(str).str.split(‘ ’, expand = True)

 Data_Frame = pd.concat([Data_Frame[‘Time’], Date_Time, Data_Frame[‘Heart Rate’]], axis = 1)

 Data_Frame.columns = [‘TimeCode’,‘Date’,‘Time’,‘HR’]

 Data_Frame = Data_Frame.set_index(‘TimeCode’)

 print(Data_Frame)

 #Plot the HR Data to View it

 # import matplotlib.pyplot as plt

 # plt.plot(Data_Frame[‘Time’], Data_Frame[‘HR’])

 # plt.show()

 #Step 1: Remove HR < 40 bpm or HR > 200 bpm

 Data_Frame = Data_Frame.drop(Data_Frame[(Data_Frame.HR < 40) | (Data_Frame.HR > 200)].index)

 def TimeString(N_Minutes):

 if N_Minutes < 0:

 Hours = 0

 Minutes = 0

 Hours = int(N_Minutes/60)

 Minutes = int(N_Minutes % 60)

 if Hours < 10:

 Output_String = str(0)+str(Hours)+’:’+str(Minutes)+’:00’

 else:

 Output_String = str(Hours)+’:’+str(Minutes)+’:00’

 return(Output_String)

 # Step 2: Get min HR from 10 p.m. and 7 a.m.

 #Get the RHR start and end times

 RHR_Start = TimeString(0)

 RHR_End = TimeString(420) #420 minutes = 7 a.m.

 RHR_Section1 = Data_Frame.between_time(RHR_Start,RHR_End)

 RHR_Start = TimeString(1320)

 RHR_End = TimeString(1439) #420 minutes = 7 a.m.

 RHR_Section2 = Data_Frame.between_time(RHR_Start,RHR_End)

 RHR1 = RHR_Section1[‘HR’].min()

 RHR2 = RHR_Section2[‘HR’].min()

 RHR = min(RHR1, RHR2)

 print(RHR)

 useMinThrougth = False

 if(np.isnan(RHR)):

 print(‘Using Min from throughout the day’)

 RHR = Data_Frame[‘HR’].min()

 useMinThrougth = True

 ##Step 3: Calculate the HRR

 MaxHR = 200

 Data_Frame[‘HRR’] = (Data_Frame[‘HR’]-RHR)/(MaxHR-RHR)*100

 #Pull the waking day out and look for HRR

 #Looking for missing time frames:

 MinuteData = Data_Frame.resample(‘1T’).mean()

 MissingMinutes = MinuteData[‘HR’].isna().sum()

 print(‘MissingMinutes: ‘,MissingMinutes)

 WholeDay = MinuteData.between_time(TimeString(420),TimeString(1320))

 print(WholeDay)

 MVPA_Threshold = 50

 filt = WholeDay[‘HRR’] > MVPA_Threshold

 Daily_MVPA_Minutes = len(WholeDay[filt])

 print(‘Daily MVPA (mins): ‘,Daily_MVPA_Minutes)

 #Get the group specific times

 if(Group == 2):

 SchoolDay = MinuteData.between_time(‘08:20:00’,‘14:45:00’)

 print(SchoolDay)

 SchoolDayMins = len(SchoolDay[SchoolDay[‘HRR’]>MVPA_Threshold])

 DataNotCorrupt = len(SchoolDay[SchoolDay[‘HR’]>−100,000])

 SchoolDayRecMins = DataNotCorrupt

 Recess1 = MinuteData.between_time(‘10:00:00’,‘10:20:00’)

 Recess1_MVPA = len(Recess1[Recess1[‘HRR’]>MVPA_Threshold])

 Recess2 = MinuteData.between_time(‘12:30:00’,‘12:50:00’)

 Recess2_MVPA = len(Recess2[Recess2[‘HRR’]>MVPA_Threshold])

 Recess3 = MinuteData.between_time(‘12:10:00’,‘12:30:00’)

 Recess3_MVPA = len(Recess3[Recess3[‘HRR’]>MVPA_Threshold])

 else:

 SchoolDay = MinuteData.between_time(‘08:40:00’,‘15:10:00’)

 SchoolDayMins = len(SchoolDay[SchoolDay[‘HRR’]>MVPA_Threshold])

 DataNotCorrupt = len(SchoolDay[SchoolDay[‘HR’]>-100000])

 SchoolDayRecMins = DataNotCorrupt

 print(“TIPO: “,type(SchoolDayRecMins))

 Recess1 = MinuteData.between_time(‘10:10:00’,‘10:30:00’)

 Recess1_MVPA = len(Recess1[Recess1[‘HRR’]>MVPA_Threshold])

 Recess2 = MinuteData.between_time(‘12:10:00’,‘12:30:00’)

 Recess2_MVPA = len(Recess2[Recess2[‘HRR’]>MVPA_Threshold])

 Recess3 = MinuteData.between_time(‘15:15:00’,‘15:25:00’)

 Recess3_MVPA = len(Recess3[Recess3[‘HRR’]>MVPA_Threshold])

 #Generate output

 #append the file to a CSV File

 Ouput = ({

 ‘Participant Code’:Participant_Code,

 ‘File’:File,

 ‘Group’:Group,

 ‘Resting HR’:RHR,

 ‘Whole Day Missing Mins’:MissingMinutes,

 ‘School Day Minutes Recorded’:SchoolDayRecMins,

 ‘Whole Day MVPA’:Daily_MVPA_Minutes,

 ‘School Day MVPA’: SchoolDayMins,

 ‘Recess 1 MVPA’: Recess1_MVPA,

 ‘Recess 2 MVPA’: Recess2_MVPA,

 ‘Recess 3’:Recess3_MVPA,

 ‘Use if’:useMinThrougth

 })

 Output_DF = pd.DataFrame(Ouput, index = [0])

 OutputFolder = ‘C:/Users/mafer/Desktop/Recess20min’

 os.chdir(OutputFolder)

 #Write the file if the results file does not exist, otherwise append to the existing file

 if not os.path.isfile(‘Resultados.csv’):

 Output_DF.to_csv(‘Resultados.csv’, index = False)

 else: # else it exists so happened without writing the header

 Output_DF.to_csv(‘Resultados.csv’, mode = ‘a’, header = False, index = False)
